# Deep Learning Utilization in Agriculture: Detection of Rice Plant Diseases Using an Improved CNN Model

**DOI:** 10.3390/plants11172230

**Published:** 2022-08-28

**Authors:** Ghazanfar Latif, Sherif E. Abdelhamid, Roxane Elias Mallouhy, Jaafar Alghazo, Zafar Abbas Kazimi

**Affiliations:** 1 Department of Computer Science, Prince Mohammad Bin Fahd University, Khobar 31952, Saudi Arabia; 2 Department of Computer Sciences and Mathematics, Université du Québec à Chicoutimi, 555 Boulevard de l’Université, Québec, QC G7H 2B1, Canada; 3 Department of Computer and Information Sciences, Virginia Military Institute, Lexington, VA 24450, USA; 4 Department of Computer Engineering, Virginia Military Institute, Lexington, VA 24450, USA

**Keywords:** deep learning, transfer learning, plant leaf disease detection, rice leaf disease detection, convolutional neural networks, VGG19

## Abstract

Rice is considered one the most important plants globally because it is a source of food for over half the world’s population. Like other plants, rice is susceptible to diseases that may affect the quantity and quality of produce. It sometimes results in anywhere between 20–40% crop loss production. Early detection of these diseases can positively affect the harvest, and thus farmers would have to be knowledgeable about the various disease and how to identify them visually. Even then, it is an impossible task for farmers to survey the vast farmlands on a daily basis. Even if this is possible, it becomes a costly task that will, in turn, increases the price of rice for consumers. Machine learning algorithms fitted to drone technology combined with the Internet of Things (IoT) can offer a solution to this problem. In this paper, we propose a Deep Convolutional Neural Network (DCNN) transfer learning-based approach for the accurate detection and classification of rice leaf disease. The modified proposed approach includes a modified VGG19-based transfer learning method. The proposed modified system can accurately detect and diagnose six distinct classes: healthy, narrow brown spot, leaf scald, leaf blast, brown spot, and bacterial leaf blight. The highest average accuracy is 96.08% using the non-normalized augmented dataset. The corresponding precision, recall, specificity, and F1-score were 0.9620, 0.9617, 0.9921, and 0.9616, respectively. The proposed modified approach achieved significantly better results compared with similar approaches using the same dataset or similar-size datasets reported in the extant literature.

## 1. Introduction

Rice is one of the most consumed foods globally as it is a main source of diet for many countries, including the most populated countries such as China, India, Pakistan, and others. The classification of Rice is under the class Orza type, which includes within that family other grain foods such as wheat, corn, and cereal. The reason why it is popular is that it is rich in supplements, minerals, and nutrition. It is estimated that it is a basic diet choice for more than three billion people [1]. Rice is a very general term because there are many types of rice around the globe and even the way they are grown varies as well. However, it should be mentioned that all rice plants share some commonalities in their development which are specifically three phases of development before harvest. A total of 15% of agricultural farm areas around the world are used for rice farming [2].

The main production of rice is in the east of India and Pakistan. Recently, there has been a noticeable reduction in rice production for various reasons. One of the main causes is rice plant disease or maladies. One of the most unwanted maladies is what is referred to as sheath blight, leaf blasts, and brown spots because they greatly affect rice production or grain quality. The maladies, though different, in effect, share the commonality of having spots on the plant leaves. Like many diseases, early detection can reduce or prevent the associated damage. The fundamental issue is the absence of constant observation of the plants. Other factors can be that farmers new to the field are not attentive and cognizant of the diseases that can occur to the plants and their seasons. Normally, these maladies can infect plants at any time. Yet, constant observation of plants and their growth period can restrain disease contagion [3].

Manually patrolling the vast rice field daily by farmers is an impossible task due to the vast sizes of the farms, and even, if possible, a human cannot look at each plant individually and examine it. Having farmers routinely check on rice plants daily, even if possible, would be a costly task, prone to human error, causing damage to rice plants in their path, and many other factors that will end up causing more harm than good. Classifying or diagnosing an issue is a very challenging task to be performed physically, as it involves different parameters to be observed, such as conditions, surroundings, and so forth. With the advancement of technology, one of the newest trends that researchers are looking into is the use of Artificial Intelligence (AI) and Machine Learning (ML) to assist farmers and researchers in various fields of agriculture in the early detection of rice plant disease [4,5,6]. The ongoing improvement in digital image processing and recognition methods has made it feasible and easier to detect infected crops and classify the disease that a crop has [5,6]. However, AI and ML alone will not be enough; thus, some researchers propose the use of drone technology, the Internet of Things (IoT), and cloud computing, among others, to have a complete system that can properly assist farmers in achieving good results and reducing cost [7]. However, still, the main component would be a highly efficient ML algorithm, technique, or process that can detect and properly diagnose rice disease. Therefore, researchers are still in search of the optimal ML solution for plant disease detection and diagnosis. Though research has been performed in this field recently, however, the optimal and most accurate solution is still an open research topic, and researchers are constantly working towards this goal. Researchers in agriculture are looking into the use of machine learning in plant breeding in vitro culture [8,9], stress -phenotyping [10], stress physiology [11], plant biology [12], plant–pathogen interaction [13], and plant identification [14].

The motivation for a system that can assist rice farmers in the early detection of rice disease is very clear from the above, which will not only increase production and quality but also reduce cost, which will benefit both the farmers and consumers. Technology research performed in the field of agriculture is mainly concerned with the enhancement of production and quality. The case has been made to what sets the rice plant aside from others in the field of agriculture, which requires special attention from researchers to target rice plant diseases and assist in the prevention of early detection. Research in this field is imperative, whether for the rice grains consumed by three billion people or other agricultural products that are as significant or even more popular.

The main aim of this work is to develop a system utilizing novel optimized ML and deep learning (DL) techniques that will accurately detect, classify, and diagnose rice disease automatically without human intervention. The ultimate aim is also to propose novel methods that can achieve higher diagnostic accuracy than other techniques in the extant literature that use similar datasets or similar-size datasets.

The rest of this work is organized as follows: Section 2 details the literature with regards to machine learning, deep learning in the field of agriculture, mainly rice disease, and other areas where machine learning is used. The proposed method is detailed in Section 3. Section 4 shows all the experimental results with discussion. We conclude in Section 5 and highlight the future work.

## 2. Recent Studies

Image processing is one of the main recipes in ML algorithms for the correct classification of images into their respective classes based on common features. ML algorithms usually consist of three phases: preprocessing, feature extraction, and classification. Classifiers are divided into either supervised or unsupervised algorithms. Recently, DL algorithms have been heavily used in research where proposed images are input into DL algorithms that extract features and classify images. Both ML and DL algorithms are used to tackle research problems in various fields. In Education [15], healthcare [16,17], smart cities [18], and all other areas relevant to humans. The ultimate goal is to automate tasks usually performed by humans with the added value of these tasks being performed by machines.

In [6], the authors propose the use of the Support Vector Machines (SVM) classifier for the classification of three rice crop diseases; brown spots, false smuts, and bacterial leaf blight. They proposed the extraction of features using Scale-Invariant feature transform (SIFT), Bag of Word (BoW). They additionally proposed the use of K-means clustering and Brute-Force (BF) matcher followed by SVM for classification. They used a dataset of 400 images gathered from various sources, including the American Psychopathological Society (APS), Rice Knowledge Bank (RKB), and Rice Research Institute (RRI). They reported an average accuracy of 94.16%, recall of 91.6%, and precision of 90.9%. However, their dataset was extremely small, especially when proposing multiclass classification, and SVM is a classifier that is susceptible to overfitting. In [19], the authors propose a deep convolutional neural network (CNN) for the recognition of rice blast disease. They used a dataset of 5812 divided equally between infected and non-infected rice plants, which are publicly available. Their method uses CNN for feature extraction and SVM for classification, and they reported an average accuracy of 95.83% for binary classification. In [20], the authors propose the use of image processing in controlling and monitoring rice disease. They target four rice diseases, namely rice sheath, rice brown spots, rice blast, and rice bacterial blight. They propose the use of engineered features based on shape and color. They also propose the use of standard classifiers such as k-Nearest Neighbor (k-NN) and Minimum Distance Classifier (MDC) for classification. They use a dataset consisting of only 115 images for these diseases and divide the dataset into 30% testing and 70% training. They reported an overall accuracy of 87.02% for k-NN and 89.23% for MDC. Their dataset is relatively small for multiclass classification, and they do not address the overfitting problem in their work. In [21], the authors propose ML techniques for rice leaf disease. They address three main types of rice leaf diseases, namely bacterial leaf blight, leaf smut, and brown spot. They use the dataset provided in [4], which consists of 120 images divided equally among the three diseases. For classifiers, they proposed traditional classifiers, which are Decision Tree (DT), Logistic Regression (LR), Naïve Bayes (NB), J48 DT, and K-nearest neighbor (K-NN). They reported an accuracy of 97.9% when using the J48 DT. This result is not surprising due to the limited size of the dataset. In [4], the authors propose the segmentation of the infected portion of the leaf using k-mean clustering and extracting the features based on texture, shape, and color. They used SVM and reported an average accuracy of 93.33% on training data and 73.33% on testing data. In [22], the authors use color features for rice plant disease classification. They analyzed 14 color spaces and extracted four color features from each channel with a total of 172 features. They used a dataset that consisted of 619 images with four classes: rice blast, bacterial leaf blight, healthy leaves, and sheath blight. They then used seven different classifiers to test their method, which are LR, Random Forest (RF), DT, NB, K-NN, SVM, and discriminant classifier (DC). They report the highest accuracy using SVM, with an average accuracy of 94.65%.

A detailed review of AI and ML methods for rice disease detection is performed in [23]. They review various methods in AI, ML, and even deep learning strategies for rice disease recognition due to the importance of the rice plant globally. In [24], the authors propose a faster region-based CNN (Faster R-CNN) for the detection of rice leaf disease in real time. Their proposed Faster R-CNN is enhanced with the use of the regional proposal network (RPN). RPN is able to precisely locate the object location and thus generate the candidate regions. They used both publicly available datasets and generated their dataset as well. They had a combined 2400 images divided into 500 images for hispa, 650 images for brown spots, 600 images for rice blast, and 650 images for healthy leaves. Concentrating on three classes: hispa, brown spot, and rice blast; they recorded an accuracy of 99.17%, 98.85%, and 98.09%, respectively. The healthy rice leaf was accurately identified with an average accuracy of 99.25%. In [25], the authors propose the use of CNN for detecting and identifying rice leaf disease. Their study included six classes of rice disease, namely ragged stunt virus disease, bacterial leaf streak, narrow brown spot, brown spot, bacterial leaf blight, and blast. They used pre-trained models such as Mask RCNN, YOLOv3, RetinaNet, and Faster RCNN. They used a dataset of 6330 images. They reported that YOLOv3 achieved the best average precision of 79.19%. In [26], the authors propose the use of models from ANN and Deep Neural Networks (DNN) for feature-based datasets and CNN for image-based datasets for the classification of rice grains because there are different rice grains. Though they do not necessarily target rice disease, their study is relevant to rice grains of various types because they can detect healthy leaves from their varieties. They collected 75,000 images, 15,000 for each of the five different varieties of rice in their study, which are Karacadag, Jasmine, Ipsala, Basmati, and Arborio. They additionally formed a feature-based dataset by extracting 106 features from the image-based dataset set, which includes 90 color features, four shape features, and 12 morphological features. They report a grain average classification accuracy of 100% for CNN, 99.95% for DNN, and 99.87% for ANN. In [27], the authors propose the use of the deep recurrent neural network (Deep RNN or DRNN) trained using their proposed RideSpider Water Wave (RSW) and enhanced by integrating the RWW in the spider monkey optimization (SMO). They used a publically available dataset [28], which consists of three classes of diseases, namely bacterial leaf blight 100 images, blast 80 images, and brown spot 96 images. They performed segmentation after preprocessing using segment network (SegNet) and extracted features from the segments: texture features, CNN features, and statistical features. They reported that their proposed RWS-based DRNN achieved the highest accuracy of 90.5%. In [29], the authors proposed an attention-based depthwise separable NN with Bayesian optimization (ADSNN-BO) for the detection and classification of rice disease. Their proposed algorithm is based on the MobileNet structure combined with an augmented attention mechanism. Bayesian optimization is mainly used for tuning the hyper-parameters of the system. They used a dataset consisting of 2370 images [30] divided into 503 images of healthy leaves, 779 leaf blasts, 565 rice hispa damage, and 523 brown spots. They reported a test accuracy of 94.65%. In [31], the authors propose new CCNN-based inception with Residual Networks (ResNet) v2 combined with an optimal weighted extreme learning machine (WELM) they refer to as the CNNIR-OWELM-based algorithm for rice disease classification. Their integrated system combines IoT for capturing images and histogram segmentation for segmenting the infected regions. Then features are extracted using the deep learning inception with ResNet v2. The WELM is optimized using the flower pollination algorithm (FPA) for the classification. They used the publicly available dataset [32], which of 38 images of leaf smut, 37 images of brown spots, and 40 images of bacterial leaf blight. They reported an average accuracy of 94.2%. A review of deep learning algorithms for rice leaf classification, detection, and diagnosis is presented in [33]. As mentioned earlier, ML and DL are now used to automate complex tasks usually performed by specialized individuals with the added benefit of reducing cost and eliminating human errors. The section highlighted some of the recent work performed for rice leaf detection and diagnosis using DL and ML. However, after developing a robust algorithm for rice leaf detection, it should be integrated with other technologies such as IoT, cloud computing, and real-time processing to make them applicable in real-life scenarios similar to the work presented in [34,35]. Table 1 summarizes the latest studies on rice disease classification with their accuracies.

## 3. Research Methodology

**Figure 1:**
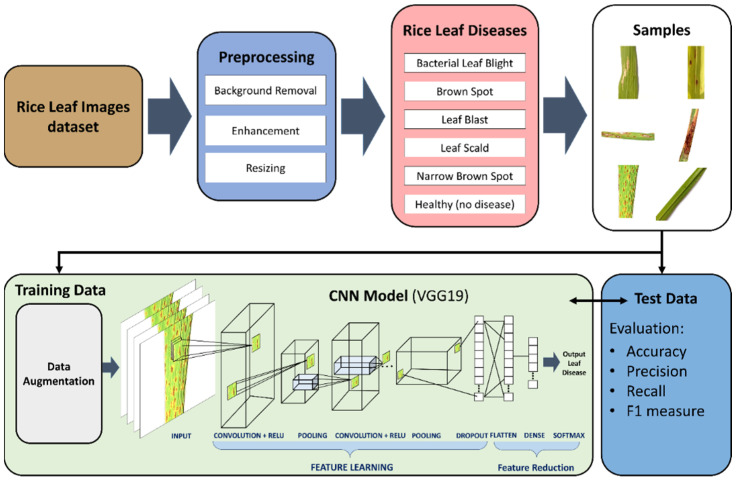
Proposed deep convolutional neural networks (CNN) transfer learning-based approach for leaf disease classification.

In this work, we propose a novel model for the classification of rice leaf disease. The proposed system as shown in Figure 1 will be able to detect and classify six distinct classes; healthy, narrow brown spot, leaf scald, leaf blast, brown spot, and bacterial leaf blight. The proposed system is one of few in the extant literature able to classify 6 distinct classes. Most papers in the extant literature contain between 2–4 classes. In the proposed deep CNN transfer learning-based approach, the images will go through preprocessing stages, where images will be subjected to background removal, resizing, and enhancement. Data augmentation is also performed to increase the size of the dataset. As discussed in the literature review, most of the papers in the extant literature use small size datasets, which can cause overfitting even though the authors did not address the overfitting problems that may occur. In this work, we apply data augmentation, which simply applies minor changes to the original images to produce new distinct images. The minor changes can include rotation, scale-in/scale-out, and translation. The features are then extracted using VGG19. The feature reduction is performed using the flatten, dense, and softmax layers in VGG19. The last layers of the VGG19 perform the classification. We evaluate our proposed approach using the following metrics; accuracy, precision, and F1-measure. The proposed deep VNN transfer learning based-approach is detailed below.

### 3.1. Experimental Data

**Figure 2:**
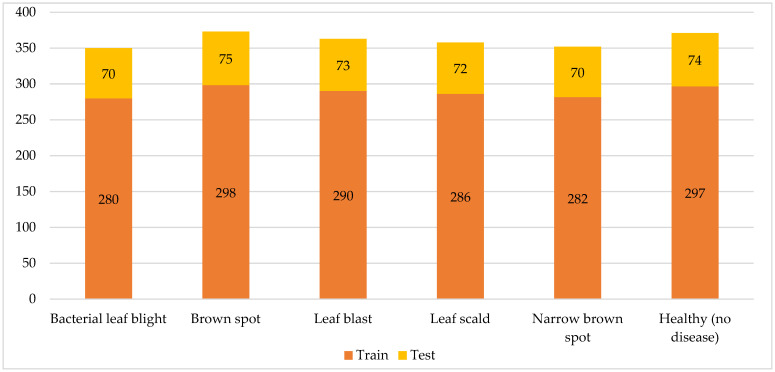
Distribution of train and test rice leaf images for different rice diseases.

The dataset used in this research includes five rice leaf diseases, bacterial leaf blight, leaf scald, brown spot, narrow brown spot, and leaf blast, along with the healthy rice leaf [39]. Figure 2 shows the distribution of train and test rice leaf images for different rice diseases. The first dataset label represents one of the most dangerous diseases that can leave a destructive impact on a growing rice crop, which is a brown spot. The disease results from a fungus named “Bipolaris oryzae”. It starts with the appearance of brownish to grayish spots in the center of the leaf, surrounded by yellow tips. As the disease progresses, the color and size of the spots might change correspondingly; however, its shape will be mostly round.

**Figure 3:**
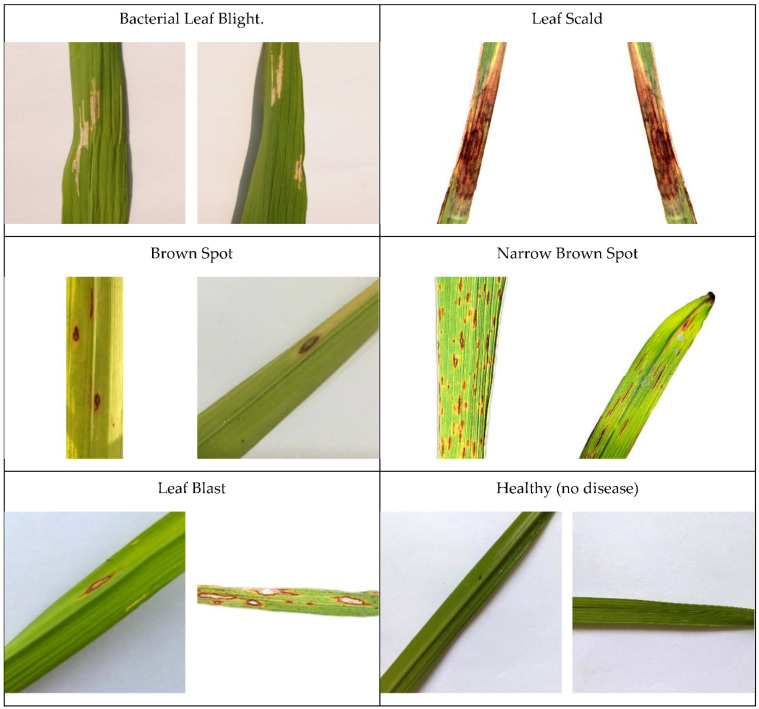
Sample Images of rice leaf diseases.

Therefore, it can develop to its extreme, in which the whole leaf color would turn yellow and die. Thus, brown spot disease leads to quantitative and qualitative losses in crops [40]. On the other hand, the healthy labeled dataset shows healthy rice with no diseases detected. Moving on to Hispa, which is a disease that initiates from an average-sized, black-colored insect named “Dicladispa armigera”. This type of insect is dangerous, regardless of whether it is an adult or a grub. This disease begins when the female insect first places her eggs separately at the abdominal part of the leaf end. After some time, when the grub comes out, the nature of the grub is that they excavate the leaf to reach the tissues in between its layers, which they feed on. Due to this excavating, the leaf turns out to be white, membranous, and therefore dies. Lastly, the dataset displays a disease that initiates from “Magnaporthe Oryzae” fungus named leaf blast. This disease leaves a destructive impact on all the segments of a rice plant that is visible above the ground. Its effect firstly shows on the leaf as white to gray marks that are bordered with red color. Their shape is typically diamond with pointy edges. As the spots enlarge, they can end up killing the whole leaf. Figure 3 shows the sample images of rice leaf diseases.

### 3.2. Preprocessing (Enhancement and Augmentation)

Image Enhancement was applied to enhance the quality of the original dataset, and augmentation was applied to increase the dataset size. Smoothing and increasing image detail results in flattening and enhancement of the contract of the images. This is performed through the manipulation of the edge-aware local contrast. By using this technique, the strong edges stay intact by defining a minimum intensity amplitude that serves as a threshold value. In this paper, the threshold was set as 0.15, and the enhancement value of 0.5. An anisotropic diffusion filter is used in the process for smoothing the contrast. Shifting the zero-frequency component to the center of the spectrum is achieved using the Fourier transform.

It is extremely important in any machine learning research that the researchers try to ensure that overfitting is prevented. In [41], the authors proposed several approaches to address such issues, including L1 regularization, L2 regularization, stochastic pooling, dropout technique, early stopping, and augmentation. In this paper, we propose the use of data augmentation, which will increase the dataset size, which in return reduces the chances of overfitting. Data augmentation is a simple process of applying minor changes to the original images to produce new images. We use the following methods in this work to include rotation, translation, and scale-in/scale-out approaches. These are three simple methods that produce new images that are closely related to the original images. Rotation, from its name, indicates a process of rotating the original image. We rotate the images by +15 to −15 degrees. Scale-in/scale-out is a zoom-in and zoom-out process. Here we scale by 105–115% for both height and width, and finally, translation means shifting the image across the x and y-axis. Here, the images are translated between −5 to +15.

### 3.3. Convolutional Neural Networks (CNN)

Convolutional Neural Networks (CNN or ConvNet), a class of deep neural networks specialized in image recognition, have developed tremendously in recent years in various fields, including agriculture. CNN uses multiple blocks of convolutional layers, pooling layers, and fully connected layers to create conceptual spatial-temporal hierarchies of features using backpropagation in an adaptive and self-optimizing manner [42]. The main idea of CNN is to build a deeper network with a much smaller number of parameters.

Like any typical neural network model, CNN is based on neurons organized in layers, starting with an initial input layer and ending with the final output layer, connected by learned biases and weights. In between are hidden layers that transform the feature space of the input to match the output with at least one convolutional layer as a hidden layer, which is required in a CNN to form patterns. On the other hand, unlike other primitive methods where feature extraction is hand-engineered, CNN does not require manual feature extraction. It can learn these characteristics automatically.

As its name implies, the convolutional layer plays a crucial role in the operation of the CNN, using adaptive kernels (number, size, and padding) that have a small size but can propagate throughout the depth of the entire network. This layer performs a convolution operation on the input layer and passes the result to the next layer and the nonlinear function such as ReLU (Rectified Linear Unit).

Furthermore, the pooling layer, known as downsampling, simply performs a dimensional reduction of the number of convolved features in the input. This action minimizes the computational power during data processing (i.e., reducing the image size by decreasing the number of pixels). Therefore, the effectiveness of the training must remain useful and accurate, and non-overfitted despite the spatial reduction. Last, the fully connected layer (FC) contains neurons that are directly connected, with no other intermediate layers. It generates a class score that is used in the classification process.

Moreover, before the training process involving the convolutional layer and the pooling layer, all the parameters used in the CCN must be fixed, while the kernel weights are learned during training, which means that a good activation function leads to a faster learning process and a reduction of the loss function quantifying the difference between the true and the predicted outputs. The weights are updated using optimization algorithms such as gradient descent or different variants of gradient descent derived from the loss function. In contrast, increasing the size of the data set as well as regularizing the data (i.e., randomly omitting some activations) results in less possibility of overfitting.

### 3.4. Fine-Tuned CNN Transfer Learning-Based Model

The key steps of training and testing require computational resources and a large amount of storage, especially when metadata is involved. Conversely, the fine-tuning technique of the transfer learning-based model is a useful approach to adjusting resource usage by performing feature extraction using “network surgery.” Fine-tuning modifies the actual architecture and optimizes memory usage. Building and validating a CNN model by selecting the most appropriate parameters using trial-and-error methods to determine the learning rate, number of layers, number of nodes, etc., can indeed be a complicated task.

There are several methods for fine-tuning the CNN, including updating the architecture, re-training the model, and partially freezing layers to use some of the weights already trained. Principally, the process of fine-tuning consists of four main steps:

1. The CNN model is pre-trained.
2. The last output layer is truncated, and all model designs and parameters are copied to generate a new CNN.
3. The head of the CNN is replaced with a set of fully connected layers. Then the model parameters are initialized randomly.
4. The output layer is trained from scratch, with all parameters fine-tuned based on the initial model.

Visual Geometry Group (VGG) is a deep CNN architecture with multiple layers. VGG-16 and VGG-19 consist of 16 and 19 convolutional layers, respectively [43]. These architectures are constructed using very small convolutional filters to increase the network depth. Both VGG16 and VGG19 take as input an image of size 224 × 224 with three color channels. The input is passed to convolutional layers with the smallest possible receptive field of size 3 × 3 and max-pooling layers. In the VGG network, the ReLU activation function then reduces the training time of the first two VGG sets having conv3-64 and conv3-128, respectively. ReLU is a feature used in AlexNet, an extension of LeNet, to speed up the learning process, apply max-pooling instead of average, reduce the size of the network by overlap pooling filters, reduce overfitting, and improve generalization. The architecture of AlexNet consists of 8 layers: 5 convolutional networks and 3 FC layers. The last three sets with the same activation function use conv3-256, conv3-512, and conv3-512, respectively.

A max-pooling layer follows each set of convolutional layers with stride 2 (number of pixels shifts across the input matrix) to maintain spatial resolution, resulting in a 2 × 2 window. Furthermore, the number of channels used in the convolutional layers differs between 64 and 512. DenseNet, an extension of Res-Net, adopts multilayer feature concatenation for all subsequent layers, which facilitates the training process of deep networks by reducing the number of parameters in the learned model. This avoids direct summation of the preceding layers, which decreases the efficiency of the model. In this study, the DenseNet-201 architecture with 201 deep layers is executed, which contains 4 dense blocks with sets of 1 × 1 and 3 × 3 convolutional layers. Each dense block is followed by a transition block with a 1 × 1 convolutional layer and a 2 × 2 pooling layer, except for the last block, which is followed by a classification layer with a 7 × 7 global average pool. This last block is followed by a fully connected network with 4 outputs.

The VGG19 network has 16 convolutions with ReLUs between them and five max-pooling layers. The number of filter maps of the convolutions starts at 64 and grows until 512. After the convolutions, there is a linear classifier made-up of three fully-connected (FC) layers with a 50% dropout between the first FC and second FC layers. The first two have 4096 features while the last one has 6. Learning Rate 1 × 10^−4^, batch size 200.

In addition, the GoogleNet architecture allows the network to choose between multiple convolutional filter sizes in each block by using inception modules and operating at the same layer, which improves computational efficiency. The architecture consists of 22 layers of parameters and 9 stacked inception modules, giving a total of 27 layers. GoogleNet takes as its base layer the inception module, which is then stacked on top of the other layers, where parallel filtering of the input layer from the previous layer is applied. SoftMax loss functions work as classifiers for the 4 classes.

**Figure 4:**
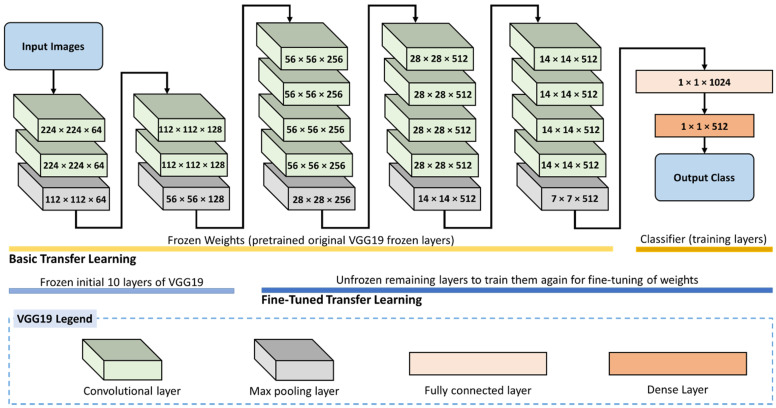
Fine-tuned transfer learning for the VGG19 model for rice leaf disease identification.

In this work, two levels of fine-tuning were applied. Figure 4 shows the proposed fine-tuned transfer learning for the VGG19 model for rice leaf disease identification. The first consists of freezing all layers of feature extraction and unfreezing the FC levels at which classification is performed. Conversely, the second stage involves freezing the first layer of feature extraction and unfreezing the last feature extraction along with the fully connected layers. This second stage requires more training and time; nonetheless, it is excepted to give better results. In this latter level, only the initial 10 layers of VGG16 are frozen, while the remaining layers are re-trained for fine-tuning.

### 3.5. Evaluation Metrics for the Experiments

There are various metrics to evaluate different machine learning methodologies’ performance. The most common seven metrics, accuracy, precision, recall, specificity, F1 score, loss function, and confusion matrix, are used to evaluate the proposed method’s performance [44]. The recognition accuracy of the framework is determined by mean Average Precision (mAP). It is the basic measurement used to perceive objects for every class. Mean Average Precision is calculated by dividing the number of correct detections for every one of the classes over the aggregate of several correctly detected and the number of incorrectly detected images. Mean average precision is observed for different types of parameters. These parameters include minimum batch size, the picture scale that is additionally the short edge of the picture, and the scaled input picture’s maximum pixel size. Mean average precision is calculated for each class/object detected in the image. Average precision calculates the average precision over 0 to 1 esteem for recall value using the following formula.

(1) $$P=\frac{No\;of\;True\;detection}{No\;of\;True\;detections+No\;of\;False\;detections}$$

The loss function is another metric that plays a major role while evaluating CNN’s performance. The classification loss function is used when you have to predict from a limited set of outcomes called classes. Cross-Entropy that is also known as logarithmic loss, is a classification loss function.

Table 2 shows the equations and explanations for the various metrics used in this work. It should be noted that $T_{P}$ represents True Positive, $T_{N}$ represents True Negative, $F_{P}$ represents False positive, and $F_{N}$ represents False Negative.

## 4. Results and Discussion

Initially, the experiments were performed using the well-known CCN models for the non-normalized dataset, normalized and augmented dataset, and non-normalized augmented dataset. The well-known CNN models explored include GoogleNet, VGG16, VGG19, DenseNet201, and AlexNet. DenseNet201 achieved the best accuracy among the well-known CNN models. Table 3 shows the results. For the non-normalized dataset, DenseNet201 achieved an average accuracy of 89.86%. For the normalized augmented dataset, DenseNet201 achieved an average accuracy of 88.33%, and for the non-normalized augmented dataset, DenseNet201 achieved an average accuracy of 83.41%. For the non-normalized dataset, GoogleNet achieved the lowest average accuracy of 83.87%, while in the normalized augmented dataset and the non-normalized augmented dataset, AlexNet achieved the lowest average accuracy of 82.38% and 79.72%, respectively.

After applying the transfer learning-based optimized weights, the experiments were repeated using the same well-known CNN models. The results are shown in Table 4. Using the non-normalized dataset, VGG19 achieved the highest average accuracy of 96.01%, and GoogleNet was the lowest performing with an average accuracy of 89.63%. For the normalized augmented, VGG16 achieved the highest average accuracy of 94.76%, while GoogleNet achieved the lowest average accuracy of 86.9%. When using the non-normalized augmented dataset, VGG19 achieved the highest average accuracy of 96.08%, while AlexNet achieved the lowest average accuracy of 85.71%.

**Figure 5:**
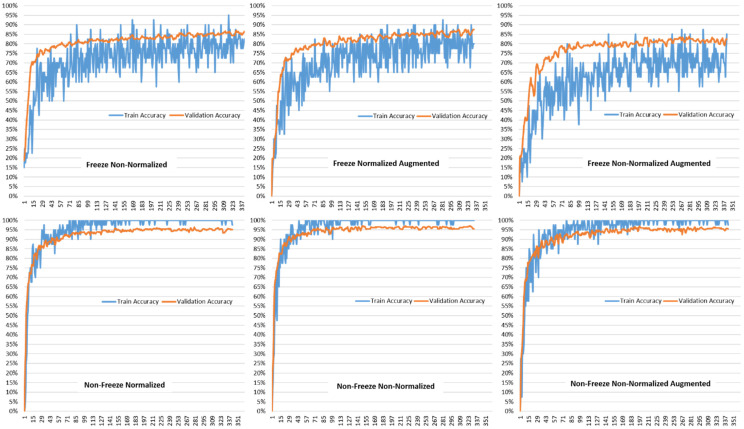
Comparison of Train Accuracy and Validation Accuracy for different model setups for VGG19-based Transfer Learning.

Figure 5 shows the training and validation accuracies for the various model setups using the VGG19-based transfer learning model proposed in this paper. It can be seen that the training and validation accuracies start with accuracies in the range of 80–85% for the freeze non-normalized, freeze normalized augmented, and freeze non-normalized augmented. The range then increases to between 90–95% for non-freeze normalized, non-freeze non-normalized, and non-free non-normalized augmented data. In all cases, the training and validation accuracies have the same trend, which shows that the over-fitting problem was accounted for. Since the validation accuracies are following the trend of the training accuracies, this proves that the model is working as designed for new data with the same accuracy for the data that it was trained for.

**Figure 6:**
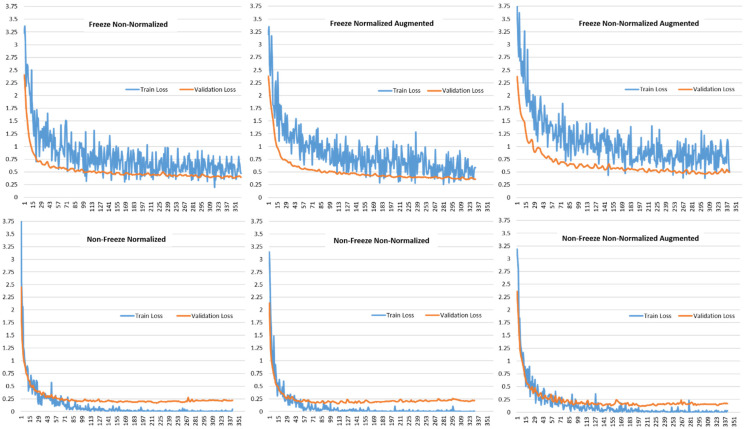
Comparison of Train Loss and Validation Loss for different model setups for VGG19-based Transfer Learning.

Figure 6 shows the validation loss and training loss for the various model setups using the VGG19-based transfer learning approach. It can be observed that the loss curves follow the same trend of continuously decreasing and ultimately reaching a stability point with a small difference between the training and validation losses. This shows that the proposed approach is a good fit that is neither over-fitting nor under-fitting. The continuous decrease in the loss accuracies towards zero and then reaching stability with a small gap between the training and validation trend is an indication of a good fit approach.

**Figure 7:**
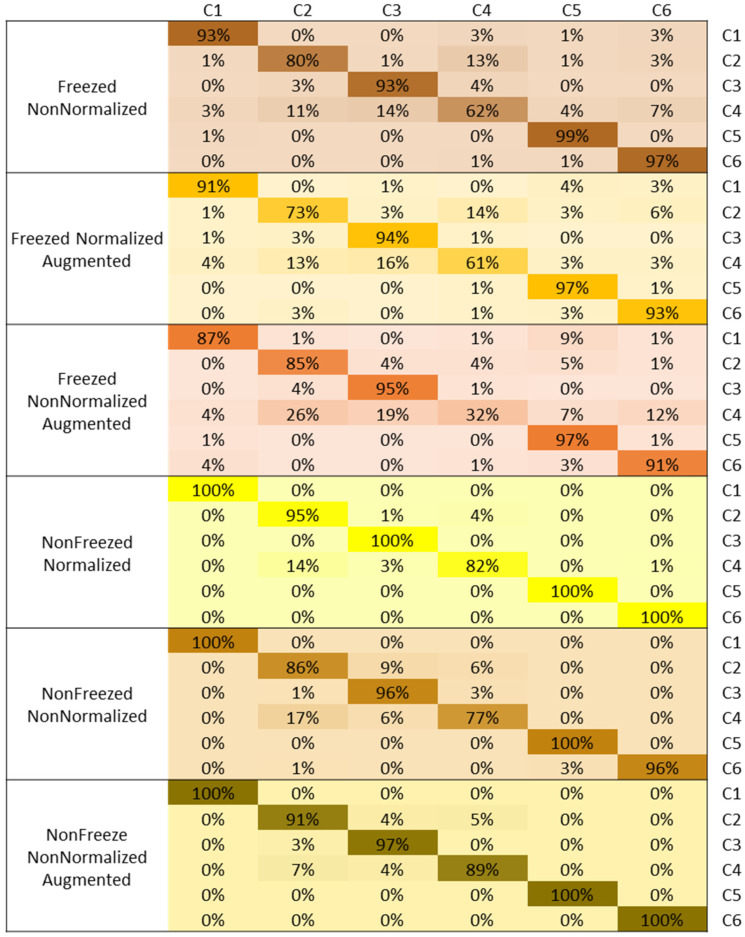
Confusion Matrix based comparison for different rice diseases identification for VGG19 with different model setups.

The confusion matrix comparison of the rice disease class identification and diagnosis for the various models using the VGG19-based transfer learning is shown in Figure 7. The confusion matrix shows that the classification accuracies are high for all classes; however, C4 shows higher misclassification in all models. C2 is showing the next higher misclassification in all models. However, in general, all classes show high classification accuracies.

The complete dataset used in this work, along with its enhanced and augmented images, has not been used by other researchers; thus, a direct comparison of results is not possible. However, Table 1 shows a summary of studies performed on other datasets, most of which are considered small datasets compared to the dataset used in this work. It should be noted that most of these works did not target classifying the number of rice diseases that are targeted in this study. The approach proposed in this study produced higher performance accuracy than those reported in the extant literature, even though we are targeting a larger number of classes of rice disease, which makes the problem more complex. This study also accounts for problems of overfitting and underfitting, which is not a claim that other previous studies can account for, especially those that use small datasets.

Once a system can be deployed within rice fields to take real-time images and process them immediately or send them to a home base where the images can be processed and proper decisions are taken, only then can we measure the exact benefit of such a system. Training is required for only one individual who will be operating the system as opposed to training a large number of farmers to visually diagnose rice plant diseases. As these systems are researched, we will be able to someday achieve an optimal solution of a complete system that can diagnose all rice diseases and be deployed for field tests.

## 5. Conclusions

Leaves are among the main parts of plants where diseases are visibly apparent. Different diseases affect the leaves in different ways that make them distinct from each other. Rice plants are very important because it is a source of food for over half the population of the world. Diseases that infect rice plants greatly affect the quality and quantity of rice produced. It is estimated that rice disease can cause 20–40% production loss annually. The manual detection of these diseases requires disease knowledge from farmers and requires extensive work to visually observe vast farmlands with individual rice crops to achieve the task of early diagnosis. This seems to be an impossible task, and even if it was possible, this would be a very expensive task that would end up increasing the price of rice for consumers. The alternative would be to find an automated method that will be able to perform early detection and decrease the cost. With the recent advances in computing, computer vision technology is gaining momentum. The features of rice leaf disease that are visually distinct can be used as features for computer vision-based systems. In this paper, we propose a modified approach of a VGG19-based transfer learning method for the accurate detection and diagnosis of six classes, which include the healthy rice leaf meaning five rice diseases can be accurately diagnosed based on leaf images. The rice leaf dataset consists of healthy leaves and five diseases, including narrow brown spots, leaf scalds, leaf blasts, brown spots, and bacterial leaf blight. The highest average accuracy using the modified proposed method is 96.08% using the non-normalized augmented dataset. The corresponding precision, recall, specificity, and F1-score were 0.9620, 0.9617, 09921, and 0.9616, respectively. Fitted onto drone technology and combined with IoT technology, the system is able to diagnose rice disease in real time.

Future work will include a complete drone technology-based IoT Technology based deep learning system that can be practically tested in real-life real-time scenarios. In addition, work will continue in our pursuit of the optimal deep learning technique able to diagnose all the rice leaf diseases that exist. In addition, and related to the field of agriculture, we plan to explore other plant leaf diseases of plants that are similarly important to humankind.

## Figures and Tables

**Table 1:** Summary of different latest studies on rice disease classification with their accuracies.

Reference	Method	Dataset Used	Performance (Accuracy %)
Sowmyalakshmi et al. (2021) [31]	CNNIR-OWELM-based deep learning	115 images	94.2%
Wang et al. (2021) [29]	attention-based NN with Bayesian optimization	2370 images	94.65%
Bashir et al. (2019) [6]	SVM image processing-based technique	400 images	94.17%
Liang et al. (2019) [19]	Convolutional Neural System (CNN).	5808 samples	95%
Prajapati et al. (2018) [4]	K-means clustering and Support Vector Machines	NA	73.33%
Kaur et al. (2018) [36]	k-NN and SVM	NA	95.16%
Ramesh et al. (2018) [37]	L*a*b, HSV and Texture Features with ANN classifier	300 images	90%
Lu et al. (2017) [38]	Convolutional Neural Networks	500 images	95.48%
Joshi et al. (2016) [20]	Minimum Distance Classifier (MDC) and k-NN	115 images	89.23%

**Table 2:** Metric Equations and explanation.

Metric	Equation	Measure
Accuracy	$\frac{T_{P}+T_{N}}{T_{P}+T_{N}+F_{P}}$	A measure of the ratio of all correct classifications to the total number of the classifications
Precision	$\frac{T_{P}}{T_{P}+F_{P}}$	The ratio of the true positive cases over the total classified positive cases
Recall	$\frac{T_{P}}{T_{P}+F_{N}}$	(Sensitivity) The measure of the proportion of the actual positive cases that were classified correctly
Specificity	$\frac{T_{N}}{T_{N}+F_{P}}$	The measure of the proportion of the actual negative cases that were classified correctly
F1-Score	$\frac{2T_{P}}{2T_{P}+F_{P}+F_{N}}$	The harmonic mean of the precision and recall

**Table 3:** Comparison of experimental results using different well-known CNN architectures with their trained weights.

	CNN Model	Accuracy	Precision	Recall	Specificity	F1_score
Non-Normalized	GoogleNet	83.87%	0.8373	0.8404	0.9677	0.8379
	VGG16	88.71%	0.8885	0.8889	0.9774	0.8835
	VGG19	87.10%	0.8674	0.8725	0.9742	0.8681
	**DenseNet201**	**89.86%**	**0.9005**	**0.9005**	**0.9797**	**0.8986**
	AlexNet	86.18%	0.8764	0.8629	0.9722	0.8554
Normalized Augmented	GoogleNet	85.24%	0.8492	0.8524	0.9705	0.8480
	VGG16	87.14%	0.8723	0.8714	0.9743	0.8677
	VGG19	85.00%	0.8465	0.8500	0.9700	0.8454
	**DenseNet201**	**88.33%**	**0.8795**	**0.8833**	**0.9767**	**0.8797**
	AlexNet	82.38%	0.8588	0.8238	0.9648	0.7975
Non-Normalized Augmented	GoogleNet	82.03%	0.8235	0.8219	0.9640	0.8158
	VGG16	82.72%	0.8515	0.8279	0.9653	0.8202
	VGG19	81.11%	0.8128	0.8120	0.9622	0.7920
	**DenseNet201**	**83.41%**	**0.8460**	**0.8364**	**0.9668**	**0.8368**
	AlexNet	79.72%	0.8100	0.8004	0.9594	0.7899

**Table 4:** Comparison of experimental results using transfer learning-based optimized weights with well-known CNN architectures.

	CNN Model	Accuracy	Precision	Recall	Specificity	F1_Score
Non-Normalized	GoogleNet	89.63%	0.8964	0.8976	0.9792	0.8967
	VGG16	95.62%	0.9570	0.9571	0.9912	0.9570
	VGG19	96.01%	0.9626	0.9614	0.9921	0.9609
	DenseNet201	94.24%	0.9433	0.9435	0.9885	0.9431
	AlexNet	92.63%	0.9306	0.9272	0.9852	0.9251
Normalized Augmented	GoogleNet	86.90%	0.8721	0.8690	0.9738	0.8675
	VGG16	94.76%	0.9501	0.9476	0.9895	0.9475
	VGG19	92.38%	0.9255	0.9238	0.9848	0.9233
	DenseNet201	92.86%	0.9277	0.9286	0.9857	0.9280
	AlexNet	88.81%	0.8868	0.8881	0.9776	0.8857
Non-Normalized Augmented	GoogleNet	86.64%	0.8681	0.8677	0.9732	0.8639
	VGG16	94.93%	0.9529	0.9499	0.9898	0.9503
	VGG19	96.08%	0.9620	0.9617	0.9921	0.9616
	DenseNet201	88.71%	0.8983	0.8897	0.9774	0.8887
	AlexNet	85.71%	0.8584	0.8597	0.9714	0.8555

## Data Availability

The data used in this research are acquired from publicly available at: https://www.kaggle.com/datasets/adefiqri12/riceleafsv3 (accessed on 24 May 2022).
